# Inventory of Toxic Plants in Morocco: An Overview of the Botanical, Biogeography, and Phytochemistry Studies

**DOI:** 10.1155/2018/4563735

**Published:** 2018-05-03

**Authors:** Hanane Benzeid, Fadma Gouaz, Abba Hamadoun Touré, Mustapha Bouatia, Mohamed Oulad Bouyahya Idrissi, Mustapha Draoui

**Affiliations:** Laboratoire de Chimie Analytique et de Bromatologie, Faculté de Médecine et de Pharmacie, Université Mohamed V, Rabat, Morocco

## Abstract

Since they are natural, plants are wrongly considered nondangerous; therefore people used them in various contexts. Each plant is used alone or in mixture with others, where knowledge and the requirements of preparation and consumption are not mastered. Thus, intoxications due to the use of plants have become more and more frequent. The reports of intoxications made at the Antipoison Center and Pharmacovigilance of Morocco (ACPM) support this finding, since the interrogations suffered by the victims show that the use of plants is practiced irrationally, anarchically, and uncontrollably. Faced by the increase of these cases of poisoning in Morocco, it seemed necessary to investigate the nature of poisonous plants, their monographs, and the chemicals responsible for this toxicity.

## 1. Introduction

Since immemorial time, the human has used plants, first to feed himself and then to heal himself. He learned, step by step, to recognize the edible plants from poisonous plants, using some of them for warlike, criminal, and magic purposes or to facilitate fishing or hunting. This relationship has become more and more intimate and diversified, for the satisfaction of nutritional (food, condiments, and drinks), aromatic (perfume), medicinal, and ornamental needs.

**Figure 1:**
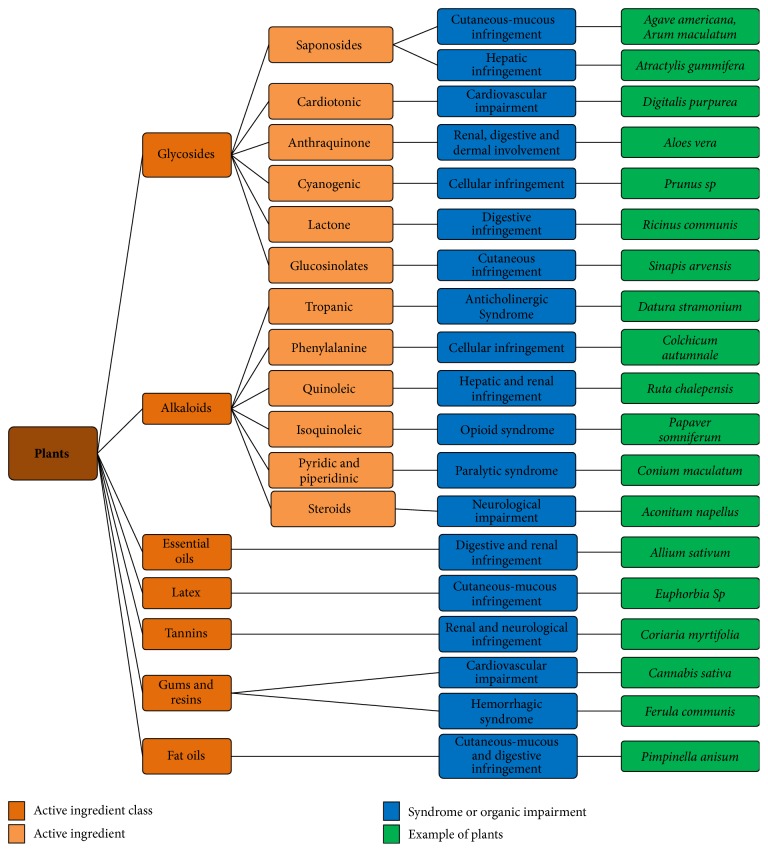
Representative diagram of the classification of plants by organic attack according to the active principle; with some examples from the CAPM database [1].

Thus, a plant is considered toxic when it contains one or more harmful substances for humans or animals and whose use causes various disorders more or less serious or even death (Figure 1) [1, 2].

This definition must take into account the place of cultivation of the plant and the time of its collection, the active principle of the toxic plant which is distributed throughout the plant or preferentially in one or more of its parts, root, berries, or leaves, and finally the notion of the administered dose [3, 4].

**Figure 2:**
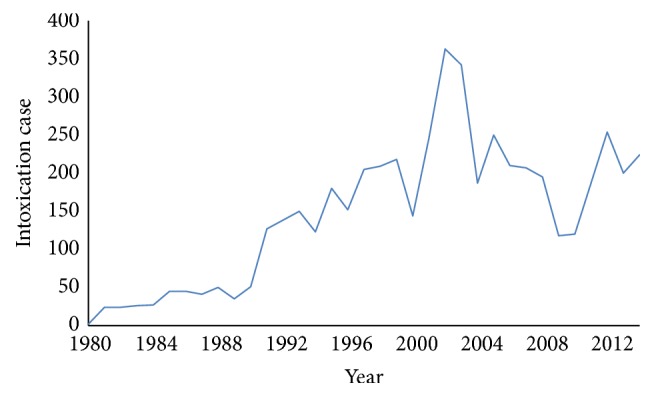
Annual evolution of PPTP intoxication cases, ACPM, 1980–2015 [6, 7].

The Antipoison Center and Pharmacovigilance of Morocco (ACPM) are sounding the alarm about the frequency and severity of poisoning due to the plants, as shown in Figure 2, taken from its annual report.

Thus, we thought it is necessary to study the nature of these poisonous plants and their monographs.

## 2. Methodology

The poisoning reported in the ACPM shows that the use of plants and products of the traditional pharmacopoeia (PPTP) is far from being negligible. Indeed, faced with the abundance of Moroccan flora, a large slice of the population prefers to heal naturally by herbal preparations alone or in mixtures. However, PPTP may contain powerful chemical compounds, responsible for adverse effects and significant toxicity and hence the need for continuous vigilance [5, 6].

According to the ACPM study from 1980 to 2008, plant poisoning accounted for 5.1% of all poisonings and ranked 7th after drugs (28.3%), gaseous products (23.3%), food (15.2%), pesticides (11.2%), household products (5.8%), and industrial products (5.2%) [1, 7].

So, we used the classification of ACPM for toxic plants in Morocco, to try to develop the monographs in order of gravity according to Table 1.

Indeed, to get closer to these plants and before developing their monographs, we present in Table 2, the different nomenclatures and the families to which they belong.

## 3. Monographs of the Most Toxic Plants in Morocco

### 3.1. *Atractylis gummifera*

#### 3.1.1. Botanical Study

The glutton thistle is a perennial herbaceous plant with a voluminous, sweeping, fleshy rhizome 30 to 40 centimeters long. It has deeply cut leaves in spiky lobes grouped in a rosette. The pink flowers are grouped in capitula surrounded by bracts bristling with spines. After fruiting, a yellowish white latex exudes in the armpit of bracts [8]. The fruit is achene hairy, ellipsoidal, and surmounted by a white egret [9].

#### 3.1.2. Biogeography

*Atractylis gummifera* is a thistle distributed throughout the world but particularly abundant in the Mediterranean regions.

In northern Africa (Algeria, Morocco, and Tunisia) and in southern Europe (Italy, Greece, Spain, and Portugal) [8].

This species is found throughout Morocco except in desert or arid regions and Anti-Atlas [9].

#### 3.1.3. Toxic Part(s)

All parts of the plant contain the toxic principles of the glue thistle. These parts, in order to decrease concentration, are root, stem, bracts, flower, seed, and leaf. The aerial parts of the plant are the least toxic [10].

#### 3.1.4. Phytochemistry

**Figure 3:**
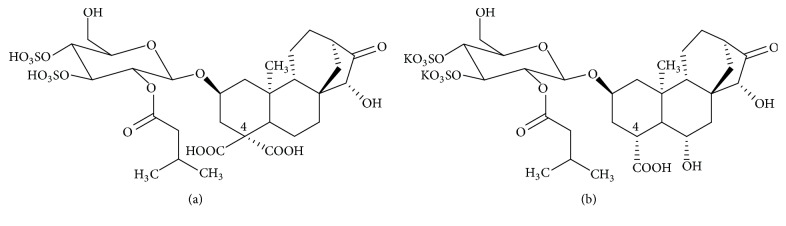
The main structures of* Atractylis gummifera* L. [14, 15].

*(1) Active Ingredient*. The toxic principles of the glutton are diterpene-derived heterosides derived from kaurene. The genome of the major compound carboxyatractyloside (ATR) (Figure 3(a)), or gummiferin, is substituted at C-4 with 2 carboxyls and at 2 and 15 with 2 hydroxyls. The saccharide part of the molecule consists of a desulfated glucose at 3′ and 4′ and acylated at 2′ by an isovaleric acid.

The other compound: atractyloside is C-4 monocarboxylic (Figure 3(b)) [8].

*(2) Chemical Structure*. See Figure 3.

The amount of ATR varies from 0.12 to 1.57% depending on the source and the season [11].

### 3.2. *Cannabis sativa*

#### 3.2.1. Botanical Study

It is a large annual plant, 1 to 2 meters high, hairy and rough, with strong odor. The stem is erect and simple. The leaves are opposite, petiolate, and palmatised, made of 5 to 7 segments toothed and acute. The flowers are green, insignificant in appearance, and dioecious [12].

The male flowers are gathered in panicles and the female flowers are grouped into compact cymes mixed with leafy bracts. The fruit is an ovoid achene [8].

Sown in May and harvested in September hemp is an excellent rotating head. Thanks to its deep and much branched roots, it improves the soil structure, increases the water capacity of the soil, and protects it from hydromorphy. Sown last among spring crops, hemp grows very rapidly from 2.5 meters to 3.5 meters between May and September.

The fruit (chennevis) is an ovoid achene. Examined under the microscope, the sheet shows on both sides numerous hairs, 1 cell, with smooth wall and curved end. Some have a bulge at the base, due to the presence of calcium carbonate crystals (cystoliths); they overlap each other like fish scales. The secretory hairs, rare in the case of the leaves, are more numerous on the bracts of the female inflorescence: multicelled pluricellular hairs often detached from its globular 8-16-cell head [13].

#### 3.2.2. Biogeography

Cannabis, one of the most common drugs on the illicit market, is grown all over the world and increasingly in local production in European countries, the United States, and Canada. Its resin form (hashish) is produced in about 60 countries and its main sources are North Africa and the countries of Southwest Asia [23].

The geographical distribution of this plant is very wide, since it can adapt to all environments. This plasticity is accompanied by variations in the general habit of the plant as well as changes in the chemical composition and in the pharmacological activity. This notion of ecological adaptation today leads to a great diversification of the species [24].

#### 3.2.3. Toxic Part(s)

The plant contains toxins; the most important ones of them are tetrahydrocannabinol, cannabidiol, and cannabinol. This is why it belongs to the category of toxic plants of therapeutic interest.

During acute intoxication, the initial phase is characterized by a period of mild euphoria and well-being and then a period of mental confusion with hallucinations and hate attacks that can lead the addiction in the worst attacks. Deadly poisoning remains exceptional [25].

#### 3.2.4. Phytochemistry

*(1) Active Ingredient*. Nearly sixty natural cannabinoids have been identified in the plant. They are non-nitrogen phenol derivatives of benzopyran. The main ones are cannabidiol (CBD), cannabinol, Δ9-trans-tetrahydrocannabinol (THC), Δ8-trans-tetrahydrocannabinol (Δ8-THC), and Δ8- and Δ9-tetrahydrocannabinolic acids.

The main psychoactive component of cannabis is THC, a major psychodysleptic, more active in its levorotatory form.

In addition to cannabinoids, cannabis smoke contains many other substances: carbon monoxide, phenols, acrolein, acetaldehyde, toluene, vinyl chloride, cresols, cyanides, acetone, ammonia, benzopyrene, benzanthracene, dimethylnitrosamine, methylethylnitrosamine, and so on, which also exhibit toxicity.

**Figure 4:**
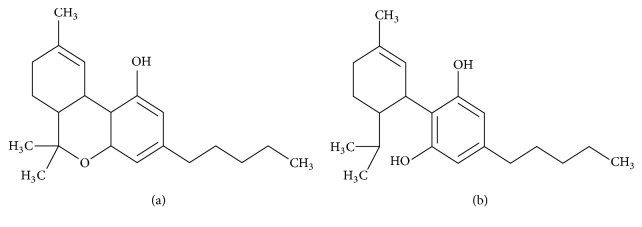
The molecules of THC (a) and CBD (b), showing their great resemblance.

*(2) Chemical Structure*. CBD has the same chemical formula and molecular weight as THC (C_21_H_30_O_2_, 314.46 g/mol), although its molecular structure is slightly different (Figure 4). Like THC and most other lipids, CBD is hydrophobic and lipophilic, which means that it does not dissolve in water and cannot be converted into an aqueous emulsion but dissolves in the fat (as well as in most organic solvents such as butane and alcohol).

In an acid medium, CBD is cyclized (it forms a new carbon ring) to become THC. In an alkaline medium (in the presence of water), CBD is oxidized to become cannabidiol-hydroxyquinone, which has been the subject of few studies to date but may have an inhibitory effect on liver enzymes (liver) that are vital for the metabolism of ingested drugs.

### 3.3. *Peganum harmala*

#### 3.3.1. Botanical Study

The harmel is a herbaceous plant, perennial, glabrous, and bushy, from 30 to 90 centimeters in height with a thick rhizome, with strong and unpleasant odor.

The erect stems, very ramous, disappear in winter; they bear alternate leaves, cut into narrow strips.

The solitary flowers, rather large (25 to 30 mm), of a yellowish white veined with green are formed of

- five green, linear, persistent sepals that extend beyond the corolla,
- five elliptical petals,
- ten to fifteen stamens with a very broad mesh in their lower part,
- the ovary, globular, resting on a fleshy disc and ending with a fruit which is a spherical capsule, with three boxes, 6 to 8 mm depressed at the top, surrounded by persistent sepals and opening with 3 or 4 valves for release the seeds.

The seeds, many, small, angular, subtriangular, dark brown in color, with an external integument, have a bitter taste; and they are harvested in summer [26].

#### 3.3.2. Biogeography

Its normal habitat is semiarid rangelands, steppe areas, and sandy soils. The plant is widely distributed in Central Asia, North Africa, and the Middle East and has been introduced to America and Australia [27].

#### 3.3.3. Toxic Part(s)

The whole plant is toxic via an alkaloid whose rate is higher in the seed (3 to 4%) than in the root or stem (0.36%) or the leaf (0.52%).

The toxicity appears for 3 g of ground seeds. The alkaloid content increases in summer, during the ripening phase of the fruit, at the time of harvest of the seed [26].

#### 3.3.4. Phytochemistry

*(1) Active Substances*. The main toxins are the alkaloids whose chemical structure is associated with an indole nucleus with a pyridinol: harmane, harmine, harmaline, and harmalol (= harmol) [28].

**Figure 5:**
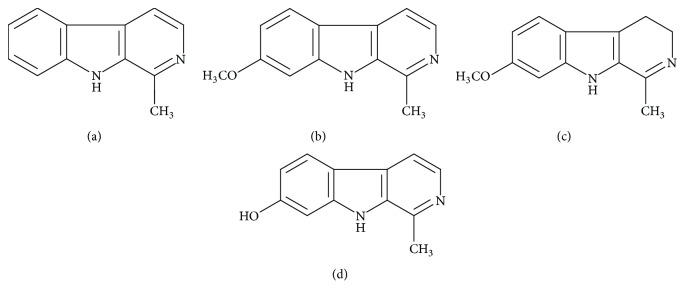
The main *β*-carboline alkaloids of* Peganum harmala* L. [16].

*(2) Chemical Structure*. The toxins have the same indole structure derived from tryptophan which associates an indole ring with a pyridine ring. The harmaline is a methoxy-harmalol and a dihydroharmine; it constitutes 2/3 of the total alkaloids of the seed; it would be twice as toxic as the harmine (Figure 5) [28].

- Harmane (a): C_12_H_10_N_2_
- Harmine (b): C_13_H_12_N_2_
- Harmaline (c): C_13_H_14_N_2_
- Harmalol (harmol) (d): C_12_H_12_N_2_.

### 3.4. *Datura stramonium*

#### 3.4.1. Botanical Study

The datura is annual, from 50 to 100 centimeters tall, ramose, and with broad leaves both hairy and somewhat viscous and repulsive odor.

The flowers in broad funnel are white, erect, shortly pedunculate, and 6 to 10 centimeters wide. The calyx is pale green, equaling almost two-thirds of the corolla. After that the flower forms a capsule as large as a walnut, erect, robust, and bristling, which opens at maturity in four valves and lets out its many black seeds, richer in toxins than the rest of the plant. It is sometimes found with purple flowers instead of white ones [12].

#### 3.4.2. Biogeography

The origin of the stramony would be American and Asian. But now it has become universal. It is mainly distributed in the Himalayas, Kashmir region of Sikkim up to 2,700 meters, in the central district hills, and south of India [8, 12, 17]. In Morocco, datura is found in all warm and temperate regions [29].

#### 3.4.3. Toxic Part(s)

All parts of the plant can be toxic, especially the seeds that are ingested. Less frequently we find the roots, leaves, flowers, or even the stem [8].

All the parts of the plant contain toxic alkaloids like hyoscyamine, scopolamine, and atropine. Because of their pharmaceutical importance, they have been studied in detail by biochemists [30].

The amount of alkaloids contained in different parts of the plant varies according to the age, the climate, and the region. The plant retains its toxicity after desiccation [30].

The toxic doses are as follows:

- A toxic dose for children is 2 to 5 g of seeds (0.1 mg/kg of scopolamine).
- A lethal dose for adults is 10 to 12 g of seeds (>2 to 4 mg of scopolamine).
- Note also that 30 to 50 seeds induce, in the majority of patients, visual hallucinations and mydriasis (with 1 g ≈ 125 seeds) [31].

#### 3.4.4. Phytochemistry

**Figure 6:**
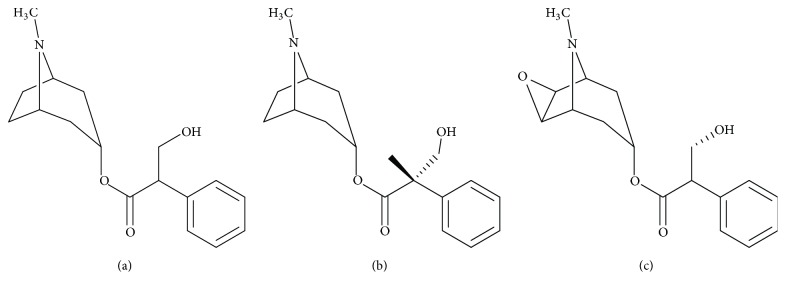
The main alkaloids of* Datura stramonium* L. [17, 18].

*(1) Active Ingredient*. All parts of the plant contain alkaloids (Figure 6): hyoscyamine (a), atropine (b), and scopolamine (c). Their quantities and proportions vary according to the species considered, the part of the plant, and the environmental conditions. Datura plants have a total alkaloid content of 0.2% to 0.6%; the third is scopolamine; two-thirds are of hyoscyamine and atropine. Young plants would be richer in scopolamine than adult plants [17].

The alkaloids are found throughout the plant, with in particular

- leaves: 0.3 to 0.5%,
- stems: 0.5 to 0.6%,
- seeds: 0.3%.

At the level of the flower, the calyx would contain 0.3% alkaloids and the corolla 0.02%.

*(2) Chemical Structure*. See Figure 6.

### 3.5. *Ricinus communis*

#### 3.5.1. Botanical Study

Castor is a dioecious, herbaceous, or arboreal species, annual or perennial depending on climatic conditions. The branching stem bears large palm-lobed leaves (5 to 12 lobes), saw-toothed, the petiole, and the underside, which are, in some varieties, purple in color. All flowers are in clusters of cymes, males with numerous stamens with branched fillet and females with tricarpel ovaries and long reddish styles. The fruit is a euphorbia capsule bristling with spikes. The seed usually has a smooth, shiny integument, usually marbled with red, black, or brown. With a fleshy prominence, the caruncle extends the upper extremity; it leaves a prominent line, clearly visible on the ventral side [8].

#### 3.5.2. Biogeography

Castor is a plant that originally developed in Egypt, Ethiopia, and India but whose culture has since spread to many other countries [32].

It is part of the Moroccan landscape; indeed, different varieties, some with large seeds, were used to fix the dunes in the region of Agadir. Morocco also once had large castor plantations whose seeds were exported to Europe for the manufacture of airplane oils and synthetic textiles. These plantations have now disappeared, but the harvests of seeds on the wild plant continue for export [33].

#### 3.5.3. Toxic Part(s)

The whole plant is enterotoxic. Castor seeds are potentially toxic. They contain one of the most harmful plant toxins: ricin.

Ricin is only present in the seed.

#### 3.5.4. Phytochemistry

*(1) Active Ingredient*. The active ingredient is a lectin: ricin, which interferes with protein synthesis.

The ricin content in castor seeds varies from 1 to 10% [34].

Castor seeds contain about 50–70% of an oil, a triglyceride whose fatty acid chains are composed of almost 90% ricinoleic acid, which is remarkable consistency. Oleic and linoleic acids are the other two significant compounds, although present in much smaller quantities: they represent, respectively, about 4 and 3% of the fatty acid chains. The other very minor compounds are palmitic, stearic, and linoleic acids, which each represent less than 1%. It is noted that pure ricin is not available [8, 32].

*(2) Chemical Structure*. Ricin belongs to the ribosome inactivating protein family (RIP) whose main molecular target is the ribosome.

**Figure 7:**
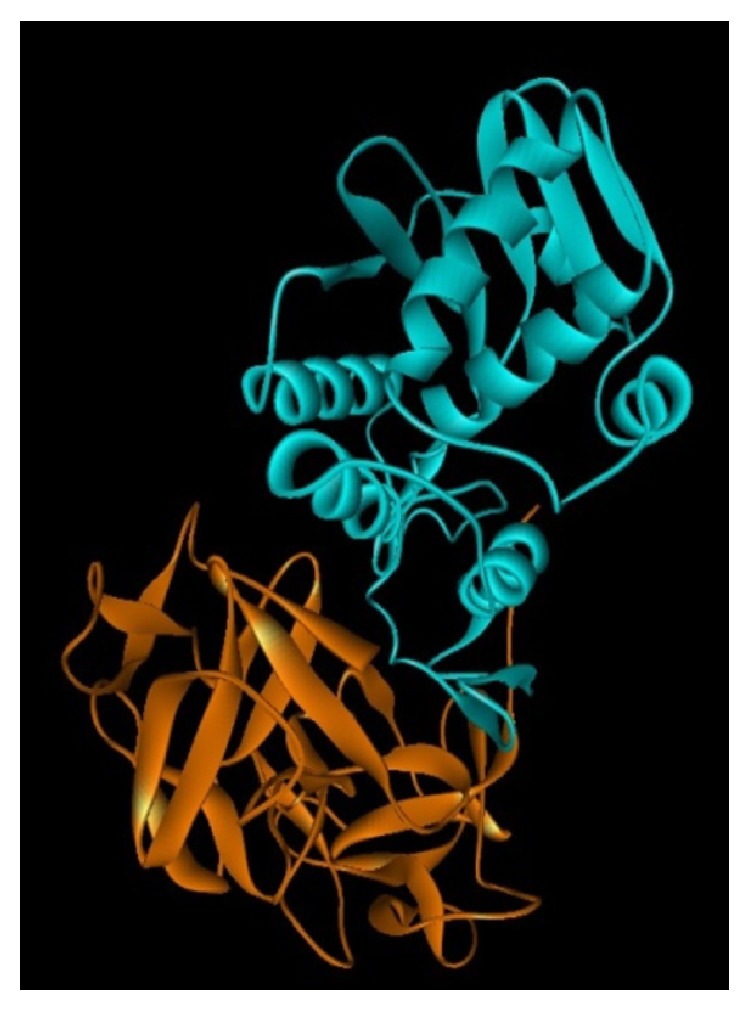
The molecular structure of ricin.

It is a glycoprotein of high molecular weight (molecular weight of about 63 kDa), composed of two subunits, A and B, of glycoprotein nature. The A chain (Figure 7, blue), an enzyme, inactivates the 28S subunit of ribosomes of eukaryotic cells and, therefore, inhibits protein synthesis.

**Figure 8:**
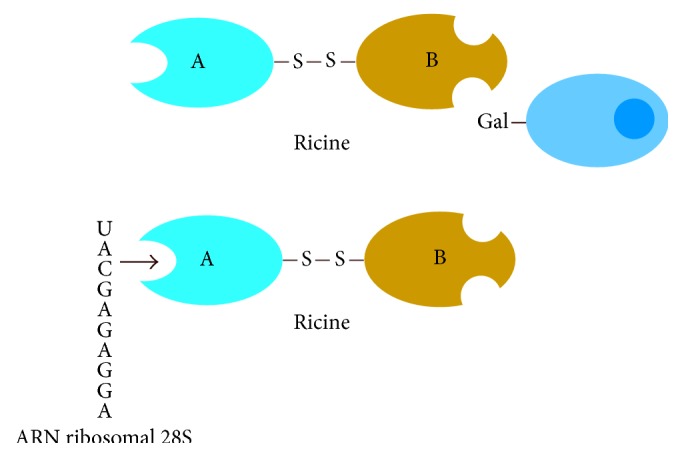
Diagram of the structure of ricin and its binding sites [19].

Meanwhile, subunit B (Figure 7 orange), a lectin, binds toxin to cell membranes having galactosylated sites as shown in Figure 8 [8, 34].

### 3.6. *Juniperus oxycedrus*

#### 3.6.1. Botanical Study

Aromatic tree or shrub can reach 8 to 10 meters high and is at an altitude of 500 to 3000 meters [35].

The leaves are opposite or whorled in needle or shell. The needles have a length from 10 to 25 mm over a width of 1.5 mm, the tip is acute, horny, and very pungent, the upper face with two whitish strips and distinct stomata [36].

The male flowers are subglobose 1 mm in diameter and are in ovoid axillary kittens or fixed in short stitches; the female flowers are globular, small with thin scales verticillate by three, welded at the base.

Male and female inflorescences are borne by separate trees [37, 38].

The fruit is a reddish ripe berry, 6 to 15 mm in diameter [39].

The cade oil or tar is the lightest fraction obtained after pyrogenation of the wood of the Cadier. It is very colored liquid (thick blackish brown) and with an empyreumatic odor [13, 40].

#### 3.6.2. Biogeography

The five taxons of juniper occupy in Morocco an area of about 244,837 ha or 5.1% of the forest area of Morocco (without the Alfa formations), including the* Juniperus oxycedrus* which is a species of Mediterranean origin, encountered especially in the mountainous regions of Morocco [33, 41, 42].

#### 3.6.3. Toxic Part(s)

It is the essential oil and oleoresins removed from* Juniperus oxycedrus* are toxic at high doses and prolonged application (carcinogenic risk) [8, 13].

#### 3.6.4. Phytochemistry

**Figure 9:**
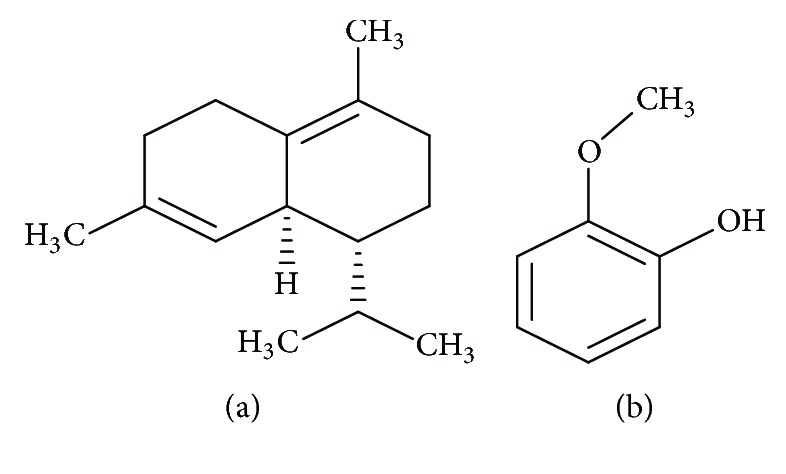
The main structures of* Juniperus oxycedrus* L. [20].

*(1) Active Ingredient*. The cade oil contains mainly carbides including cadinene (a) and guaiacol (b): a phenol (Figure 9) [40].

*(2) Chemical Structure*. See Figure 9.

### 3.7. *Lawsonia inermis*

#### 3.7.1. Botanical Study

It is shrub 2 to 4 meters in height with thin, slender branches at the ends and often becoming thorny. The leaves are simple and opposite. The small white-cream flowers are scented. The fruit is a rounded capsule.

#### 3.7.2. Biogeography

Henna is native to tropical and subtropical dry areas, including Africa, India, Sri Lanka, and the Middle East. It is commercially grown in West India, Pakistan, Yemen, Iran, Sudan, and Libya [43].

In Morocco, it is grown in several regions and there are several commercial qualities of henna according to the territories:

- Henna dukkaliya, from the Azemmour region, grown in irrigated and smoky land
- Henna filaliya, from Alnif (Tafilalet)
- Henna soussiya, cultivated everywhere in the desert and formerly in the Sequiat Al-Hamra [44].

#### 3.7.3. Toxic Part(s)

Henna is a dye authorized by the Federal Food, Drug, and Cosmetic Act (FFDCA). This is the only harmless dye. So, henna alone or mixed with natural products like lemon juice to revive the color of the tattoo or* Tamaris orientalis* to color the hair in dark black, as cutaneous application, has virtually no toxicity [45].

**Figure 10:**
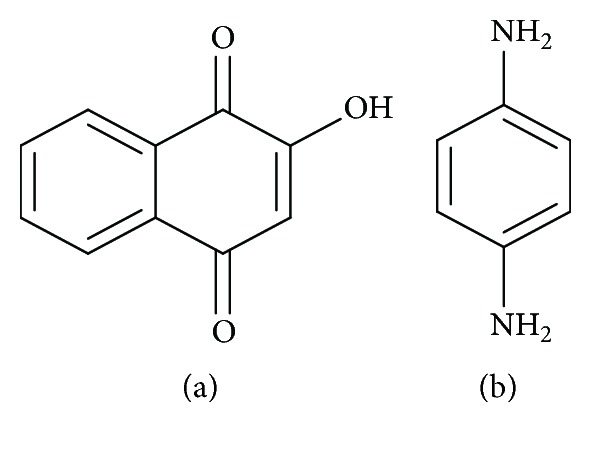
Lawsone structure (a) and PPD structure (b).

On the other hand, henna becomes very toxic when mixed with chemicals such as hexane or a mixture of alkanes (diluent) to fix the color faster and more intensely or even paraphenylenediamine (PPD) (Figure 10(b)), a synthetic chemical that produces a black color and shortens the drying time, which lasts only a few minutes to 2 hours. The tattoo thus obtained may last longer. This is commonly called, but inappropriately, the “black henna” [46, 47].

#### 3.7.4. Phytochemistry

*(1) Active Ingredient*. The main dye of henna is lawsone (2-hydroxy-1,4-naphthoquinone) (Figure 10(a)) [48].

Other constituents present are gallic acid, glucose, mannitol, fats, resin (2%), mucilage, and traces of an alkaloid. The leaves produce hennotannic acid (especially lawsone) and a green oil, a resin soluble in ether and alcohol.

The flowers produce an oil (0.01–0.02%) of brown or dark brown color, strongly scented, and consist mainly of *α*- and *β*-ionones (nitrogen compound) and a resin.

The seeds contain (5.0%) protein, (33.62%) carbohydrates, (33.5%) fiber, and (10-11%) fatty oils. They are composed of behenic, arachidic, stearic, palmitic, oleic, and linoleic acids [48].

*(2) Chemical Structure*. See Figure 10.

### 3.8. *Papaver somniferum*

#### 3.8.1. Botanical Study

It is a large annual plant, from 1 to 1.50 meters high, with a simple stem or barely branched at the top, glabrous, with leaves of a glaucous and ashen green, which seem covered with a kind of wax. The leaves of the base are petiolate, but those of the top are sessile. All are oval-shaped, irregularly divided, and glabrous.

The flowers are solitary; they present first two deciduous sepals that fall at the time of flowering and four petals pink or white, with a black tab on the base, many stamens, and a gynoecium made of about twenty carpels that weld together so as to finally form a large capsule covered by traces of stigmata. This capsule contains a large amount of small, wrinkled seeds [12].

The opium poppy has many varieties and races, differing in the color of the flowers, the seeds, the shape and size of the capsule, and the hairiness of the leaves.

Classically, we distinguish the following varieties:

- Variety* glabrum* Boiss., with purple flowers, glabrous leaves, large globular capsule (10 to 12 centimeters wide), and seeds of purplish black color
- Var.* album* DC (white poppy), white flowers with ovoid indehiscent capsule (4 to 8 centimeters in diameter) and yellowish white seeds
- Var.* nigrum *DC (black poppy or “carnation”), with purple flowers, subglobose capsule, dehiscence by pores on the edge of the stigmatic plateau, and slate-gray seeds
- Var.* stigerum* DC, with purple flowers, floral peduncles, and leaves covered with rough hairs, small capsules, and gray seeds [21, 49].

#### 3.8.2. Biogeography

This plant is native to southern Europe and North Africa. The plant was well known to many ancient civilizations such as Egyptian civilization and ancient Greece. It is also grown in western countries. It is also found in some Asian countries that clandestinely manufacture opium.

Its expansion to favorable stations of India and China did not take place until after the first millennium of the Christian era. Since its survival is incompatible with extreme heat and cold, it is found neither in the tropical and equatorial zones, nor in the southern hemisphere, nor in the boreal regions [50].

Regarding the origin of its varieties,

- the var. stigerum DC is in the semiwild state in southern Europe and grows throughout Morocco with the exception of desert regions;
- the var. Glabrum Boiss. is grown in Asia Minor;
- the var. DC album is grown in India and formerly in Iran, while var. nigrum DC is grown in Europe. These last two varieties were formerly, in Morocco, cultivated everywhere in the gardens or vegetable gardens, including the Saharan Oases [44, 49].

#### 3.8.3. Toxic Part(s)

The toxic parts are the leaf and the capsule but especially the latex that is drawn.

It is difficult to give reliable dosage indications as long as alkaloid concentrations are variable. In general, the plant is toxic at low doses [51].

#### 3.8.4. Phytochemistry

**Figure 11:**
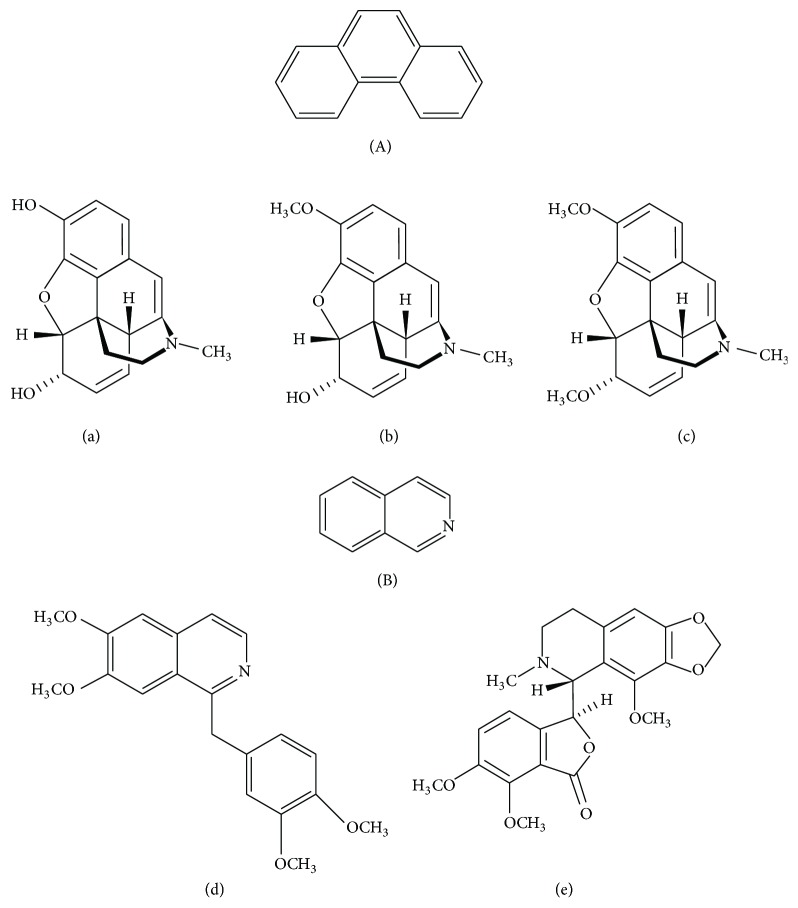
The main structures of phenanthrene and isoquinoline [21, 22].

*(1) Active Ingredient*. Opium is extracted from the capsule of the opium poppy, which contains 20 to 25% alkaloids in two groups (Figure 11):

- Phenanthrenes (A):
  1. Morphine (5 to 20%): (a): it is the main alkaloid present in opium
  2. Codeine (0.5 to 3%): (b)
  3. Thebaine (0.2 to 1%): (c)
- Benzylisoquinolines (B):
  1. Papaverine (1%): (d)
  2. Noscapine (2–10%): (e) [52]

Opium can contain 10 to 15% water. Sugars are abundant (20%) as well as organic acids: lactic acid, fumaric acid, oxaloacetic acid, and especially meconic acid (more than 5%) [13]. 

*(2) Chemical Structure*. See Figure 11.

### 3.9. *Nerium oleander*

#### 3.9.1. Botanical Study

The oleander is from 2 to 3 meters high. Its stems are glabrous and spread at the break a milky juice. The leaves are opposed by two or three, leathery, persistent, long lanceolate, and acute, with parallel veins, and tight on the stem. The flowers are pink, large, generally fragrant, and arranged in large terminal corymbs. The corolla is in saucer, with five petals welded by their base. The fruit is a follicle that sits in a cylindrical pod, and the seeds (which are rather infrequently formed) are hairy and have a feather [12].

#### 3.9.2. Biogeography

The oleander is a plant native to Europe and Africa and is commonly found in the tropical and subtropical regions of the world [53]. This plant is grown in more northern regions. It grows spontaneously on the rocky banks of rivers, sometimes even in coastal areas, usually devolved to halophilic species. It adapts to drought and is very decorative [8].

#### 3.9.3. Toxic Part(s)

All parts of the plant are toxic to humans, animals, and some insects [54]. This shrub is highly poisonous, which should not be confused with a bay leaf, used constantly as a seasoning [12].

#### 3.9.4. Phytochemistry

*(1) Active Ingredient*. The toxic principles are digitalis glycosides, present in all parts of the plant, thereof, cardenolides, which account for about 1.5% of the weight of the leaves.

**Figure 12:**
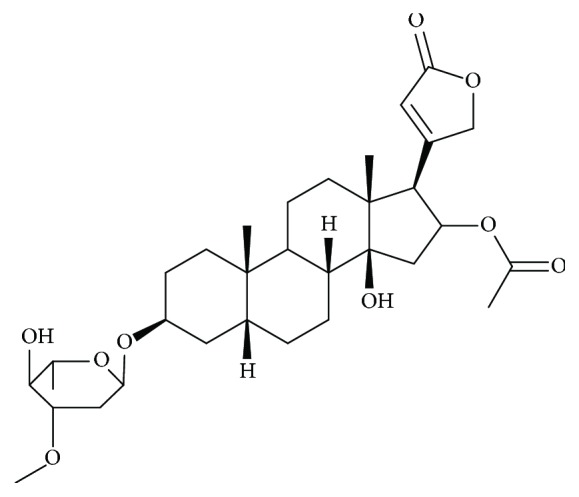
Chemical structure of oleandrin.

Oleandrine (Figure 12), the majority, is heteroside of oleandrose and oleandrigenin.

The seeds contain oleandrin and related compounds: odorosides, adigoside, and glucostrospeside [8, 55].

*(2) Chemical Structure*. Oleandrine (oleandroside) is a cardiotonic glycoside derived from an aglycone, oleandrigenin (16-acetylgitoxigenin), and a sugar oleandrose (2-deoxy-3-O-methylrhamnose), of the empirical formula C_32_H_48_O_9_. Pure oleandrin is in the form of colorless, odorless, and very bitter crystals. It is soluble in chloroform and ethanol and insoluble in water. See Figure 12.

### 3.10. *Myristica fragrans*

#### 3.10.1. Botanical Study

It is a dioecious tree, with aromatic evergreen leaves and small yellow flowers in clusters. It can reach a dozen meters high and be centenarian. Only females bear fruit. The tree bears fruit after 8 years and can continue producing for more than 60 years. The ripe fruits are picked at the time of the dehiscence of the pericarp.

The fruit is an ovoid capsule. It includes a thick, fleshy pericarp, which is eaten once confit, and a seed. The seed is surrounded by a laced aril, which gives the spice called mace. It has a thick integument and lignified shell. Nutmeg can be marketed in shell, or in albumen removed from its hull. This albumen is ruminated, which gives the many curved lines that we see in section. In the kitchen, it is used as a spice.

#### 3.10.2. Biogeography

The nutmeg is a tree producing nutmeg and is a tropical tree native to the Banda Islands in the Maluku archipelago in Indonesia and is now grown in various tropical climate regions, including the West Indies and Grenada. Production reaches 30 to 40% of world production [56].

It is formerly known since fragments of nutmeg had been found in some sarcophagi of ancient Egypt. After the discovery of the Indian route, the Arabs introduced it to Andalusia in the 15th and 16th century in Europe.

#### 3.10.3. Toxic Part(s)

All parts of the plant are toxic, a toxicity that varies depending on the part used (mace, essential oil, etc.), dose, and individual sensitivity.

In small doses, nutmeg and mace are safe (in medical or culinary use). But, in excessive doses, they are hallucinogenic and toxic.

Consuming two whole nuts can be fatal; myristicin is the substance responsible toxicity and hallucinogenic effects. In addition, isolated safrole is carcinogenic in high doses.

#### 3.10.4. Phytochemistry

**Figure 13:**
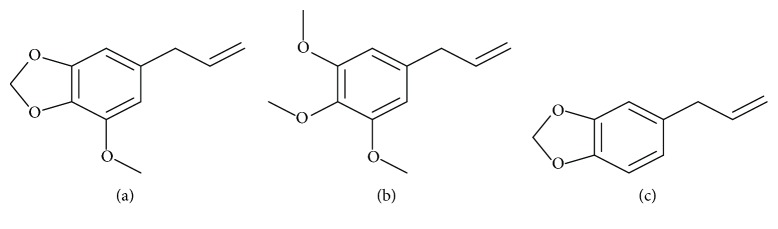
Structures of the main molecules of nutmeg.

*(1) Active Ingredient*. The active principle of this plant is myristicin (Figure 13(a)).

- Nutmeg:
  1. Essential oil (15%), comprising *α*-pinene, *β*-pinene, *α*-terpinene, *β*-terpinene, myristicin, elemicin (Figure 13(b)), and safrole (Figure 13(c))
  2. Fixed oil “nutmeg butter”: 25 to 40%, mainly, myristicin (trimyristin), terpene carbides (sabinene, pinene, limonene), palmitate, stearin, linalool, and geraniol
- Mace:
  1. Essential oil: similar to nuts, with more myristicin

*(2) Chemical Structure*. See Figure 13.

## 4. Discussion

Plant poisoning affects all regions of Morocco, with a difference in number or percentage, which can be explained by the nature of the vegetation present in each region, as well as the climate that plays an important role in the proliferation of these plants. And It does not spare any age group (newborn, infant, walker, child, teenager, adult, and senior).

From 1980 to 2013, the region of Casablanca recorded the maximum frequency of poisoning with a population of 1119 cases followed by the Meknès-Tafilalt region with 760 cases. These intoxications occurred in urban areas in 82% of cases and 18% in rural areas; the majority of intoxications occurred at home with 50.2%.

In 2015, the most represented region was Chaouia-Ouardigha 19.9%, followed by Rabat-Salé-Zemmour-Zaer 18.6% and then Meknès-Tafilalt 15.5% and Fès-Boulemane 10.6%. These intoxications occurred especially in urban areas 54.4%.

The most effective way to manage plant poisoning begins with the identification of the plant and its toxic potential. Identification is made by scientific name, common name, common vernacular names, and knowledge of environmental factors that modulate plant toxicity (growth, harvest season, and climate).

In hospitals, the precise questioning on the circumstances of intoxication makes it possible to evaluate intoxication and the delay between the ingestion of the plant and the medical consultation remains determining to save the life of a patient in state of intoxication. Finally, the toxicity must be studied for all plants, even wild plants and indoor plants, parks, and gardens that can be dangerous.

## 5. Conclusion

In Morocco, the rate of intoxication by plants is more and more frequent, because they are abundant and exist in the wild; also, we find them on sale at low price at the seasonal fresh herbalists or dried during all year. So the people use them in a variety of contexts.

This public health problem has been the subject of several outreaching programs to citizens by all means of the media. In hospitals, the security measures are applied by emergency doctors, pharmacists, and nurses bringing first aid to addicts. And lastly the ACPM has set up standardized procedures to improve the therapeutic management of this type of poisoning.

Thus, the entire population is well warned about the seriousness of this plague, which produces each year a large amount of damage which can reach to the death of the victims.

## Figures and Tables

**Table 1:** Distribution of cases of poisoning by plants according to the species involved. Extracted from the global table of the ACPM, 1980–2008 [1].

Latin name	Total	%
*Atractylis gummifera*	461	10.6
*Cannabis sativa*	441	10.1
*Peganum harmala*	201	4.6
*Datura stramonium*	156	3,6
*Ricinus communis*	101	2.3
Essential oil of *Juniperus oxycedrus*	56	1.3
*Lawsonia inermis*	31	0.7
*Papaver somniferum*	31	0.7
*Nerium oleander*	27	0.6
*Myristica fragrans*	43	1.0

**Table 2:** Families and different nomenclature of the most toxic plants in Morocco.

Plant	Family	French name	Vernacular name (or Moroccan name)	English Name
*Atractylis gummifera*	Asteraceae	Chardon à glu, Chamaeléon blanc	Addad	Thistle
*Cannabis sativa*	Cannabinaceae	Cannabis, Chanvre indien	lkif, hachich, chira, el-qenneb	Cannabis
*Peganum harmala*	Zygophyllaceae	Harmel, Rue de syrie	L'Harmel	Harmal, Syrian rue, wild rue, wild boar
*Datura stramonium*	Solanaceae	Datura, Stramoine	Chedecq ejmel, Alghita	Thorn apple, Datura, jimson weed
*Ricinus communis*	Euphorbiaceae	Ricin	Kharwâa	Castor
Essential oil of *Juniperus oxycedrus*	Cupressaceae	Cadier, genévrier oxycèdre	Katran, Getrân, Tâqqa	Cade juniper, common juniper, prickly juniper
*Lawsonia inermis*	Lythraceae	Henné, Réséda	Elhanna	Henna
*Papaver somniferum*	Papaveraceae	Pavot à opium, Pavot somnifère, Pavot noir	Kharchacha, Khechkhach,	Opium poppy
*Nerium oleander*	Apocynaceae	Laurier rose, oléandre	Defla	Oleander, Rose-bay
*Myristica fragrans*	Myristicaceae	Muscadier	Gouza (pour la noix), Bsibisa (pour le Macis de noix).	Nutmeg
